# Effect of Physical Exercise on Esports Performance in First-Person Shooter Gamers: A Six-Week Randomized Controlled Trial

**DOI:** 10.1186/s40798-025-00970-2

**Published:** 2025-12-24

**Authors:** Di Tang, Xin Zhang, Ray Wai-Keung Ho, Siu Ming Choi, Zheng Ye, Mark J. Campbell, Raymond Kim-wai Sum

**Affiliations:** 1 Department of Sports Science and Physical Education, Faculty of Education, The Chinese University of Hong Kong, Hong Kong, China; 2 Nethersole School of Nursing, Faculty of Medicine, The Chinese University of Hong Kong, Hong Kong, China; 3 Faculty of Education, Central China Normal University, Hubei, China; 4 Faculty of Education, University of Macau, Macau, China; 5 Department of Physical Education & Sport Science, University of Limerick, Limerick, Ireland

**Keywords:** Esports performance, First-person shooter, Physical exercise, High-intensity interval training, Video games

## Abstract

**Background:**

Esports has gained recognition as an official sport by multiple countries and organizations, featuring in international events like the Asian Games. Despite its growing prominence as a competitive sport, research on esports performance remains limited. Recent studies suggest that physical exercise, particularly high-intensity interval training (HIIT), can enhance cognitive functions and gaming performance. However, these studies have primarily focused on immediate or warm-up effects, leaving a gap in understanding the longer-term impact of such training interventions on esports performance.

**Results:**

This study investigates the effects of a six-week HIIT program on esports performance in first-person shooter (FPS) gamers. Healthy adult FPS gamers aged 18–29 were recruited through various platforms and randomly assigned to either an intervention group, which participated in a six-week HIIT program, or a control group. A total of 83 participants completed the study. The intervention group demonstrated significant improvements in key FPS skills, including flicking, tracking, decision-making, reaction time, and shooting accuracy. Additionally, the HIIT program enhanced VO_2max_ and sleep quality, with a moderate correlation observed between changes in VO_2max_ and reaction time over the six-week period. However, no significant enhancements were observed in mental toughness and self-regulation.

**Conclusion:**

A six-week HIIT program can improve gaming performance and overall well-being in esports players. Future research should explore the neurophysiological mechanisms behind these benefits and evaluate other physical training modalities. This study provides insights into how exercise can optimize esports performance and support a balanced lifestyle for gamers.

*Trial Registration* ChiCTR2400083252 (Chinese Clinical Trial Registry).

## Highlights

A six-week HIIT program significantly enhanced FPS gaming performance metrics, including reaction time and shooting accuracy.

HIIT improved both VO_2max_ and sleep quality in esports, with our findings revealing a moderate correlation between VO_2max_ changes and reaction time improvements.

No significant changes were identified in mental toughness and self-regulation in this study, which may be attributed to the current training protocol design.

This study supports the integration of physical training in esports, while emphasizing the need to explore neurophysiological mechanisms and optimize protocols across different gaming genres.

## Introduction

In recent years, esports has experienced remarkable growth, evolving into a prominent industry characterized by a substantial participant base and sophisticated technological infrastructure [1–4]. This rapid development has been further legitimized through its integration into prestigious international sporting events, including the Asian Games and Southeast Asian Games [5–7], while several countries, such as China, Korea, and the United States, have formally recognized it as an official sport [8]. Furthermore, the International Esports Federation reports that approximately 40 additional nations are currently in the process of evaluating esports for formal sport recognition [7].

Despite surpassing certain traditional sports in development and gaining increasing recognition as a legitimate sport, research examining esports from a sports science perspective remains notably limited, particularly in aspects of training and performance, especially when compared to established mainstream sports [9–11]. However, exploring training science and performance analysis in esports is crucial for the scientific and professional development of the field. This is particularly important given the rapid growth of professional esports tournaments and the increasing number of clubs and nations forming esports teams to participate in international competitions [11–13]. Currently, we require more scientific evidence to support the sustainable development of competitive esports, similar to what exists in traditional sports. Understanding the model and composition of esports performance and abilities is crucial for enhancing player performance and developing effective training programs [10]. Within the existing body of research on esports training and performance, there is an emerging recognition of the potential benefits of physical exercise and neuronal training for improving esports outcomes [14–17]. However, the majority of these investigations typically concentrate on the immediate enhancement effects. More empirical evidence is still needed regarding the operational mechanisms and longer-term efficacy of these training interventions [11].

Beyond the limited quantity of research, studies in esports training and performance face additional challenges. To date, there is no uniform or consensus measurement method for evaluating performance in esports [18]. It is acknowledged that esports performance involves not only physical capabilities, such as upper limb stability, hand-eye coordination, and manual dexterity, but also cognitive and psychological skills and qualities, including stress resilience, reactive capabilities, problem-solving abilities, and multitasking proficiency [10, 19]. Current performance evaluation methods generally fall into three categories. The first type of evaluation method relies on players’ rankings or scores within specific esports titles [11, 20], which provide a holistic assessment [11] but can be influenced by factors such as matchmaking systems and team dynamics, raising questions about whether these ranks truly reflect an individual’s skill level [14]. The second type of measurement analyzes performance data from specific gaming tasks or custom-designed scenarios [14, 21–23]. While these tests provide direct insights into players’ technical proficiency, they may not fully capture the psychological demands and team dynamics of actual competitive play due to their brief duration and simplified nature. The final category uses indirect methods, including psychological tests and validated assessments of abilities relevant to esports [17, 24–27]. These measures evaluate cognitive functions (e.g., reaction time, decision-making, working memory) and psychological qualities (e.g., mental toughness, self-regulation) [28, 29]. Although these tests are scientifically validated, their indirect nature may limit their ability to accurately reflect or distinguish between different levels of actual esports performance [27]. Based on expert consensus derived from a recent Delphi study, cognitive function tests and gameplay task assessments currently might represent the most appropriate and reliable methods for measuring esports performance [18].

Physical exercise has demonstrated efficacy in improving cognitive functions associated with executive control processes. These processes involve filtering redundant or inconsistent information and making rapid and accurate decisions [30]. The positive impact of physical exercise on cognition operates through neurobiological mechanisms. This form of exercise stimulates the release of various neurochemicals and signaling molecules, which may induce changes in specific regions of the brain to enhance different aspects of cognitive processing [31]. Given the active involvement of executive control processes in most video game practices, physical exercise has shown potential for enhancing esports performance [17, 32, 33]. Besides, regular exercise can provide protection against the detrimental and widespread effects of stress and anxiety on various aspects of cognition. Physical exercise has also been demonstrated to enhance reaction ability and communication skills [34–37]. These abilities are crucial factors in esports performance and training [10, 38]. In addition to the traditional exploration of the relationship between physical exercise and cognitive function, recent studies have started to directly investigate the association between physical exercise and performance indicators in video gaming or esports [21, 39–43]. Cardiorespiratory fitness and reaction time have been found to be significantly correlated in esports gamers [24]. High-intensity interval training (HIIT) is an effective, efficient exercise method that involves short bursts of intense exercise followed by rest or low-intensity exercise [44–46]. This exercise has not only been shown to boost cardiovascular health by increasing cardiorespiratory fitness in young, healthy individuals [47] but has also been demonstrated to be an efficacious approach to augmenting cognitive functions [48, 49]. Specifically, a meta-analysis demonstrated that chronic HIIT produced significant improvements in both basic information processing and executive function [49]. As a result, HIIT has become a commonly investigated method for enhancing cognitive performance [45, 50]. Research has also shown that a single bout of HIIT can improve esports performance [14, 41], specifically enhancing players’ target elimination capabilities and accuracy in multiplayer online battle arena (MOBA) games [14]. Therefore, this study aims to investigate the potential integration of HIIT into esports training by examining its effects on player performance.

In addition, it is worth noting that first-person shooter (FPS) holds a prominent position within the esports landscape. These games require players to demonstrate exceptional hand-eye coordination, reaction time, and decision-making skills [51]. Besides, shooters may be more demanding of players’ reaction time than other esports games, and the performance is more dependent on players’ reaction levels, with reaction time being one of the most essential abilities in FPS [52]. Given that HIIT has been shown to enhance neurocognitive processing speed, FPS, where split-second reactions are crucial for success, provides an ideal landscape for investigating the effects of HIIT on esports performance.

In summary, this study seeks to address current gaps in the literature and bridge the fields of exercise science and esports by investigating the impact of physical exercise on esports performance. Specifically, this study aims to examine whether a six-week HIIT intervention could improve gaming performance as well as psychological and cognitive functions in FPS players. Given the limitations of single measurement methods of esports performance, this study employed a comprehensive approach that combines gameplay skills, psychological qualities, and performance-related indicators to evaluate esports performance. Providing evidence that exercise positively influences esports performance may serve as a basis for redefining the divide between esports and physical activity, thereby encouraging greater integration. This research also has the potential to enrich the theoretical foundation of esports training and provide empirical evidence. Furthermore, it aims to promote the integration of physical exercise into the esports community, ultimately fostering a healthier and more sustainable environment for esports athletes.

## Methods

### Participants

We recruited healthy adult first-person shooter gamers for this study through the university’s mass mail system and social media platforms, including WeChat groups, Facebook, and Hong Kong online gaming forums. Additionally, the QR code for the initial online survey was displayed at offline esports activities for anyone interested in participating. The age range of participants was set between 18 and 29 years for two reasons. First, this age range represents the primary demographic of current esports participants and professional esports athletes [53, 54]. Secondly, setting the age limit at 18 is also in consideration of the regulations of the Chinese National Radio and Television Administration regarding the participation of minors in video games. Under the regulations of the National Radio and Television Administration and the implementation of the Chinese Online Game Fatigue System in games, minors have limited opportunities to engage in esports gaming [55]. Therefore, in compliance with national regulations and for the protection of minors, participants under the age of 18 were not recruited in this study. As for the determination of esports players, based on previous literature, we only recruited players who have engaged in esports games within the past six months [56]. Based on previous research examining the effects of similar HIIT interventions on esports performance [14, 42], we set an effect size (Cohen’s d = 0.6) as our benchmark. Utilizing the G*Power (ver. 3.1.9.7; Heinrich-Heine-Universität Düsseldorf, Düsseldorf, Germany) for our calculations [57], a minimum sample size of 48 participants is required to attain 80% power and maintain a significance level of 5% in a general model with repeated measures. Based on the power calculation and accounting for an anticipated 20% dropout rate, a minimum of 60 participants should be recruited for this study. In summary, our inclusion and exclusion criteria are as follows:

Inclusion criteria:

- 18-29 years old;
- regular FPS players engaging in FPS within the past six months (≥2 times/week, ≥5 hours/week) [39,54];
- healthy individuals with no contraindications to physical assessments or HIIT;
- without regular exercise habits (engaging in fewer than 2 planned exercise sessions per week) [58];
- can understand English or Chinese.

Exclusion criteria:

- self-reported history of neurological, psychiatric, or medical diseases;
- having heart disease, hypertension, musculoskeletal injuries, or any other conditions that pose a risk to engaging in physical activity;
- current intake of medications and/or recreational drugs that could affect the central nervous system and/or the ability to learn;
- a regular user of Aim Labs (described in detail in the Measurement section).

During the recruitment process, we conducted a preliminary screening of potential candidates through an online questionnaire survey based on our inclusion and exclusion criteria.

### Experimental Design

**Fig. 1:**
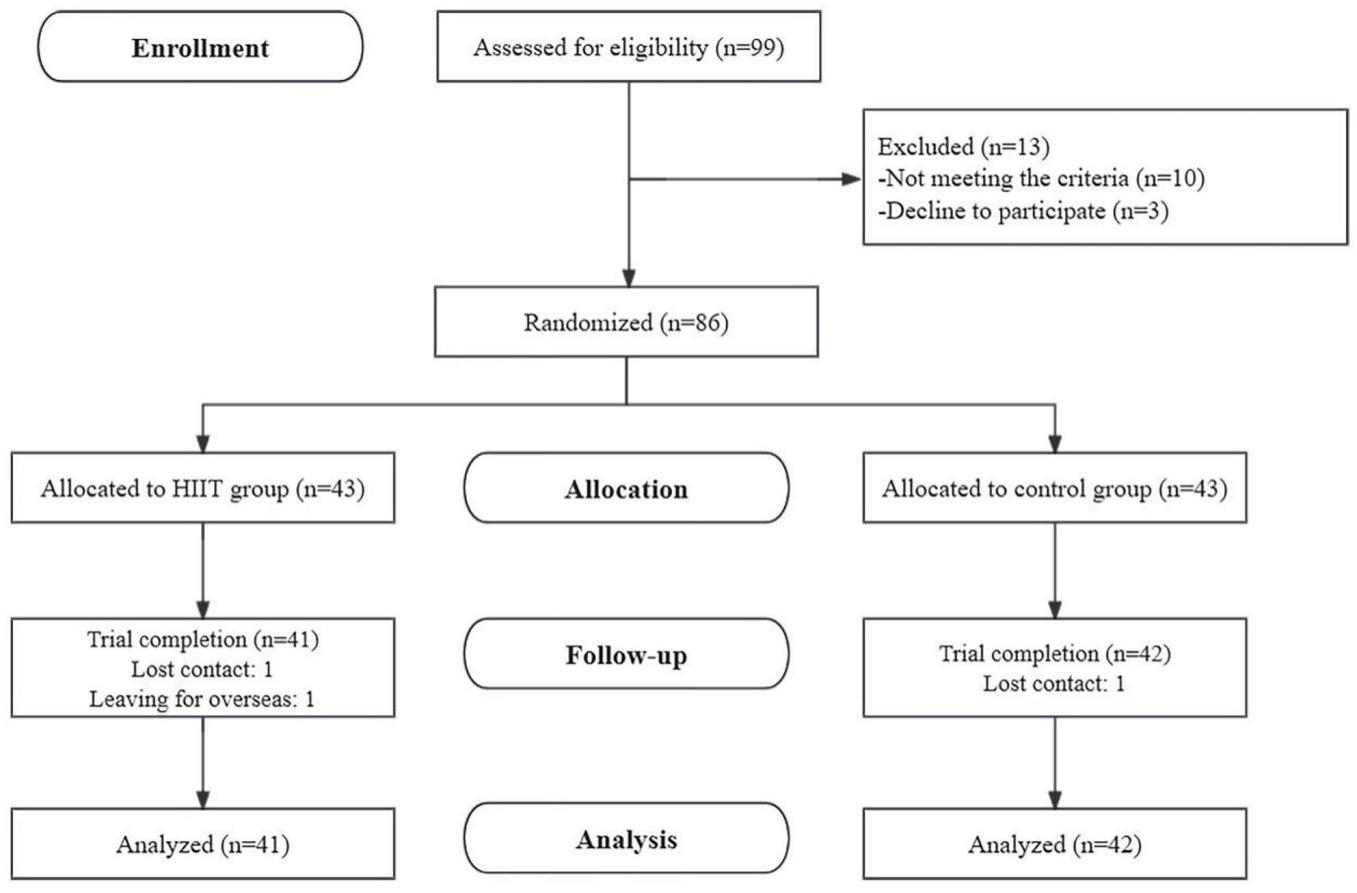
Flow chart of the experimental procedure

The protocol of this study was registered at the Chinese Clinical Trial Registry (ChiCTR2400083252). This study was designed as a randomized controlled trial. Participants were randomly allocated into two groups (1:1 ratio): intervention and control groups. A computer-based randomization tool (https://www.randomizer.org) was utilized to facilitate random allocation. The entire allocation process was conducted by a staff member who did not participate in the study. Following both online and offline recruitment, a total of 86 applicants passed the initial screening process. However, one participant withdrew from the study due to personal reasons, and two others were unable to be contacted during the process. Consequently, the data analysis ultimately included 83 participants. This number satisfied our previously estimated minimum sample size requirement. In scenarios requiring assessors to utilize measurement tools for evaluating metrics, we engaged testers who were completely blind to the participants’ group assignments to conduct the assessments. For metrics tested via computer, our assessors served merely to facilitate and oversee the process, with the computer’s saved data serving as the definitive record to ensure data objectivity. This approach has been demonstrated to be reasonable in the previous esports performance assessment [14]. The intervention group received a 6-week HIIT exercise program, while the control group did not receive any intervention. Both groups reported their physical activity, sleep patterns, and gaming activity. All the participants underwent a baseline test, followed by a post-test six weeks later. Figure 1 illustrates the specific experimental procedure.

### Measurement

#### Esports Performance

##### Gameplay Skill Assessment

To address the limitations of using a single type of current esports performance test, we have integrated multiple tests to assess comprehensive esports performance, aiming to explore the multifaceted effects of exercise on esports outcomes.

**Fig. 2:**
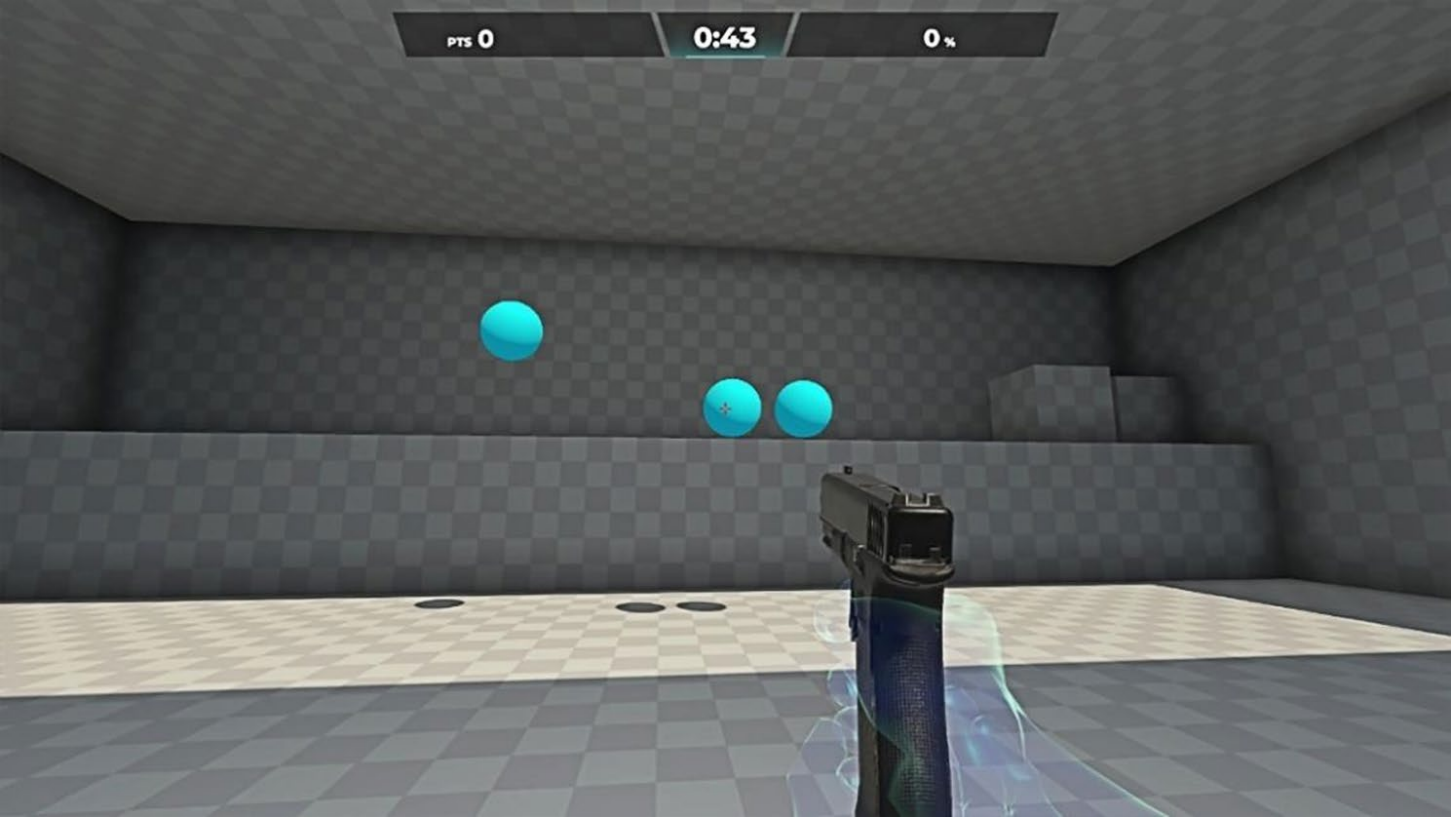
The interface during the Aim Labs testing process

First, we employed an FPS training software called Aim Labs [59] for performance measurement. Aim Labs is a freely available PC application designed with the specific purpose of enhancing aiming performance in FPS (Fig. 2). It offers comprehensive testing, training, and analysis features tailored to assess and improve players’ aiming abilities. Aim Labs has been employed in research on esports performance within the FPS gaming domain [15, 21, 60, 61]. A study has validated the use of aim training systems as reliable instruments for quantifying FPS players’ performance [62]. We selected three types of skill tests that are highly relevant to most shooting games: flicking, tracking, and decision-making. These skills are universally recognized as core elements in most FPS, rather than being specifically tailored to any particular type of shooting game. Additionally, they serve as primary indicators in the evaluation of esports performance within previous esports research [16, 38, 39, 61]. Aim Labs provides participants with detailed feedback on their performance in each test, including data on reaction times, shooting precision, and additional metrics like tracking duration in the tracking test, which were used to evaluate participants’ overall gameplay skills [15, 60].

Choosing to use a third-party shooting program for testing instead of a specific game’s custom approach has several advantages. Firstly, it ensures that all players of shooting games have the same level of familiarity with the testing program, preventing individual players from obtaining higher esports performance scores due to their proficiency in a chosen game. Secondly, Aim Labs is primarily a training program focused on practice rather than entertainment, and it is unlikely that casual players would engage in regular training with this program. By selecting this software, we can avoid the potential influence of players’ habitual use of Aim Labs during the six-week intervention, which could enhance their proficiency and subsequently affect the final performance test. Additionally, we excluded habitual users of Aim Labs during the screening phase to mitigate this issue. On the other hand, using a specific mainstream shooting game for testing would make it difficult to control the spontaneous participation of players who are loyal users of those games during the six-week intervention, leading to an increase in proficiency and potentially affecting the final game performance test.

Before the formal Aim Labs test began, each participant completed a single practice round as a warm-up session to familiarize themselves with the software, mouse, and keyboard setups. Razer Synapse software (ver. 3, Razer Inc.) [63] was used for participants to set the DPI (dots per inch). All Aim Labs tests were conducted on the same computer in our laboratory, using an identical mouse (Razer RZ01-0254), monitor (AOC 24G4; 23.8-inch, 1920 × 1080, 180 Hz), and keyboard (Razer RZ03-0263) setup. Table 1 describes the content of gaming tests. Each game test lasted 60 s. Apart from the warm-up round, each player performed the same game test twice, and their best performance was recorded as the final performance data. To prevent mutual impact, all gaming performance tests were conducted on a separate day from time-consuming questionnaires and physically demanding assessments, with a minimum 48-hour interval after the completion of other assessments.

**Table 1:** Descriptions of indicators and corresponding tests

Indicators	Description	Test	Description
Flicking	Rapidly moving the crosshair to a target and shooting accurately.	Grid shot test (T1)	Targets appear in a grid-like arrangement on the screen, and players must quickly and accurately shoot as many targets.
Tracking	Keeping the crosshair on a moving target to maintain accuracy while the target is in motion.	Sphere track test (T2)	Players are presented with a single sphere that moves dynamically across the screen, often changing speed and direction unpredictably. The primary objective for the player is to maintain their cursor focused on the moving sphere at all times, effectively tracking its movement.
Decision Making	Quickly and effectively making choices that influence the outcome of the game.	Decision shot test (T3)	A central orb appears in either of two colors: red or blue. Players are required to shoot this orb. Upon doing so, two new orbs will appear on the screen. The player must then quickly identify and shoot the orb that matches the color of the previously shot orb.
Reaction	Performing actions quickly, such as aiming and shooting.	T1 and T3	The average reaction times at T1 and T3.
Accuracy	Hitting targets precisely.	T1 and T3	The average hit rates at T1 and T3.
Mental Toughness	Resilience and the ability to persistently pursue goals, maintaining focus and determination under pressure.	Sports Mental Toughness Questionnaire	
Self-Regulation	Managing and controlling behavior, emotions, and thoughts to adapt to environmental demands and achieve personal goals.	The Self-Regulation Questionnaire	
Working Memory	Temporarily holding and processing information.	Backward Corsi Task	After observing a sequence of rectangles lighting up on the screen, participants are required to click the rectangles in reverse order.

T1: Test 1; T2: Test 2; T3: Test 3

##### Psychological and Cognitive Performance

Given that brief, single-session game tests may overlook the impact of physical exercise on the psychological and certain cognitive functions of esports players, we supplemented operational game testing with additional psychological and cognitive assessments. The psychological tests primarily focused on mental toughness and self-regulation, traits that are challenging to capture in short, non-competitive experimental gaming environments. Previous studies have demonstrated a correlation between mental toughness, self-regulation, and the proficiency of esports players [28, 29]. The Sports Mental Toughness Questionnaire (SMTQ) and The Self-Regulation Questionnaire (SRQ) were used for assessment. SMTQ is a 14-item instrument designed to determine the mental toughness levels of athletes and is divided into three sections: six items aimed at assessing confidence, four items measuring constancy, and an additional four items designed to evaluate control [64]. SRQ is composed of 63 items divided into six distinct dimensions: evaluating, triggering, searching, planning, implementing, and assessing [29, 65].

Furthermore, considering that current game testing primarily involves cognitive functions related to reaction time and decision-making, we selected a test of working memory, the Backward Corsi Task, designed to assess spatial working memory capacity. The correlation between spatial memory and gaming proficiency has been established in prior research [66, 67]. Table 1 provides descriptions of these performance indicators and corresponding testing.

#### Other Relevant Variables and Control Variables

##### VO_2max_

In previous research, VO_2max_ has been shown to correlate with cognitive function [68–70], a correlation that also manifests among esports players [24]. Therefore, this study also included VO_2max_ as one of its secondary indicators. VO_2max_ was assessed through the Progressive Aerobic Cardiovascular Endurance Run 20-m (PACER 20-m), which is a validated assessment for VO_2max_ commonly used in sports science research [71, 72]. While such field-based assessments have expected error margins due to their indirect nature, validation studies have shown these errors fall within acceptable ranges [73]. We estimated VO_2max_ from the number of completed laps in the PACER test [74].

##### Affect

In both the pre-test and post-test, participants were required to complete the Positive and Negative Affect Schedule (PANAS) [75]. The PANAS is a self-reported questionnaire that consists of two 10-item scales to measure both positive and negative affect.

##### Physical Activity

The evaluation of physical activities was conducted through the self-administered International Physical Activity Questionnaire (IPAQ) long form [76]. This specific version of the IPAQ comprises a set of 27 questions designed to ascertain the duration (in minutes) of participants’ involvement in physical activities of different intensity levels.

To ensure that the study results were not influenced by participants who might engage in unplanned additional aerobic exercise during the six-week intervention period, we excluded individuals with an established regular exercise routine from the recruitment process. This precaution was taken to maintain the integrity of the intervention’s impact on test outcomes.

##### Sleep

Participants were asked to maintain their usual sleep habits over the six weeks, and they were required to complete the Pittsburgh Sleep Quality Index (PSQI) [77] both before and after the pre-test and post-test sessions. The PSQI is a 19-item self-rated questionnaire for evaluating subjective sleep quality over the previous month. The 19 questions are combined into 7 clinically derived component scores. In this test, higher scores indicate poorer sleep quality.

##### Gaming Behavior

A simple questionnaire created by our team was used to collect information on the participants’ weekly engagement in esports gaming. During the intervention period, we also asked the players to maintain the same frequency of gaming and gaming habits as before.

#### Perception

Given that this study represents an innovative exploration into the effects of sustained and regular exercise interventions on the performance of esports players, it is venturing into relatively uncharted territory with limited prior research. After all participants completed a six-week HIIT, interviews were conducted with those who agreed to participate. The motivation for adopting this supplementary qualitative approach includes two main aspects. First, the currently available metrics for evaluating esports performance are neither comprehensive nor fully refined. The subjective feedback from the participants will provide crucial insights for further refinement of esports performance metrics. Second, the impact of HIIT on esports players is not fully understood, particularly in terms of the underlying mechanisms. Consequently, we believe that soliciting the subjective experiences and perceptions of the esports players through post-intervention interviews will provide valuable directional insights for our future research endeavors. These endeavors aim to clarify the relationship between physical exercise and esports performance, potentially enriching the substantive depth of our findings. Since our interviews primarily aimed to exploratorily investigate participants’ experiences and self-perceived changes during the intervention, we employed semi-structured interviews with open-ended questions to understand their perceived personal changes and impacts on gaming performance during the intervention period. The interviews were conducted face-to-face individually, with each session lasting approximately 20–30 min.

### Intervention

Based on previous research findings, HIIT programs lasting more than four weeks are recommended [78]. Additionally, according to the ACSM (American College of Sports Medicine) guidelines for HIIT and previous literature, programs lasting 6–12 weeks and 3–7 times a week are recommended [79, 80]. Therefore, in this study, a 6-week HIIT program (3 times a week) was chosen as our physical exercise intervention. All participants were required to complete and sign the Physical Activity Readiness Questionnaire (PAR-Q) before physical assessment and intervention to screen for any contraindications to exercise and ensure they had no risk factors that would preclude their safe participation.

From previous literature, it has been observed that a HIIT protocol involving 10 × 1-minute bouts at 80–90% of maximum heart rate (HR_max_) with 1-minute recovery periods at 40–50% of HR_max_ is deemed effective [78, 81, 82]. We employed the age-predicted equation (HR_max_ = 208 − 0.7*age) for HR_max_ prediction, which, although not without error, showed superior stability and predictive accuracy across our study’s age distribution [83, 84]. Moreover, this intensity and interval have shown significant short-term effects on esports performance in previous studies [14], and such intensity is also suitable for the participants involved in this study who did not have a regular exercise habit. A standardized 3-minute warm-up and dynamic stretching, and a 3-minute cool-down at 50% HR_max_ were added at the start and the end of the exercise. The average total duration of each training session was approximately 26 min. Real-time heart rate monitors (Polar Pacer) were used to ensure intensity during exercise sessions. In addition, a rating of perceived exertion (RPE) was employed in each training session utilizing the 6–20 Borg scale [85]. During high-intensity intervals, participants were instructed to maintain an RPE between 15 and 18, while during the low-intensity intervals, warm-up, and cool-down phases, an RPE of 11–13 was targeted [86]. All training sessions were guided by a professional fitness trainer who ensured the professionalism and safety of the entire exercise process.

Participants were allowed to undertake the training either on a treadmill or outdoors, through running, jogging, and brisk walking, provided they reached the stipulated heart rate. During the initial three sessions, participants were obliged to exercise in a laboratory under the guidance and supervision of research staff. This ensured not only a precise understanding and execution of the training regimen but also the safety of participants during subsequent unsupervised exercises. Once it was ascertained that participants could meet the training benchmarks without supervision, they were permitted to carry out their HIIT exercises at home in a free-living setting for the remainder of the intervention. HIIT has also been demonstrated to be implemented safely and successfully in a home-based environment [86]. During these home-based exercises, participants were still required to wear a Polar Pacer to track exercise intensity. The monitor recorded the exercise data throughout the training program, and with prior consent obtained from participants, we monitored each participant’s exercise data through the Polar Flow for Coach, including metrics such as heart rate, frequency, and running speed to confirm participants’ compliance with the exercise plan. This research design aims to further mitigate participant drop-out rates in long-term interventions and construct an exercise intervention model that can be implemented long-term in a living environment rather than in a laboratory setting, and the methodological approach has also been employed in prior studies [81, 87].

### Strategies to Improve Adherence

Firstly, before enrollment, we repeatedly confirmed the participants’ willingness and compliance with the study’s requirements. Secondly, dedicated researchers were responsible for monitoring the intensity and frequency of the participants’ weekly exercise through the Polar Flow for Coach system, and they repeatedly reminded and guided them to complete the six-week training plan as required. At the same time, the researchers maintained contact with the participants over the six weeks and instructed them to maintain the same gaming frequency, dietary habits, and sleep patterns as they had before the experiment. Thirdly, the participants received financial compensation in the form of a HK$200 supermarket voucher upon completion of the 6-week intervention.

### Statistical Analysis

Continuous variables were reported as means with standard deviations. The normality of the data was assessed using the Shapiro-Wilk test. The Generalized Estimating Equations (GEE) were used to determine the group-by-time effect because they do not require a distribution assumption. An exchangeable working correlation matrix was used for the GEE analysis. The normality test was performed to determine which tests to use in further data analysis. For the normally distributed data, the paired t-test was used to examine the time effect within each group, and the independent t-test was used to examine the differences between groups at baseline and post-assessment. For data not normally distributed, the Mann-Whitney U-test and the Wilcoxon Signed-Rank Test were used instead. The significance level was set at *p* < 0.05 to determine statistical significance. Pearson correlation coefficient and Spearman’s rank correlation coefficient were used based on the data distribution. Additionally, we conducted inductive thematic analysis of the interview transcripts to explore participants’ perceptions of the six-week HIIT program, allowing themes to emerge naturally from the data.

## Results

Data from 83 participants were finally analyzed. The background variables are presented in Table 2. Among the recruited players, there were no significant differences in gaming experience (*p* = 0.872, Z = − 0.161) or weekly gaming hours (*p* = 0.513, Z = − 0.655). This ensured consistency in baseline gaming levels among the players.

**Table 2:** Background variables for the two groups

	HIIT Group (*n* = 41)	Control Group (*n* = 42)
Male (n, %)	34 (82.9)	35 (83.3)
Age (years, SD)	22.15 (2.74)	21.21 (2.51)
Weight (kg, SD)	66.77 (10.62)	68.35 (14.33)
BMI (kg/m^2^, SD)	22.37 (2.85)	22.83 (3.88)
Gaming Years (years, SD)	7.37 (4.23)	7.12 (3.08)
Weekly Gaming Hours (h/week, SD)	8.89 (6.76)	8.27 (5.17)

BMI: Body mass index; SD: Standard deviation.

### Effect of the Exercise Intervention on Esports Performance

**Fig. 3:**
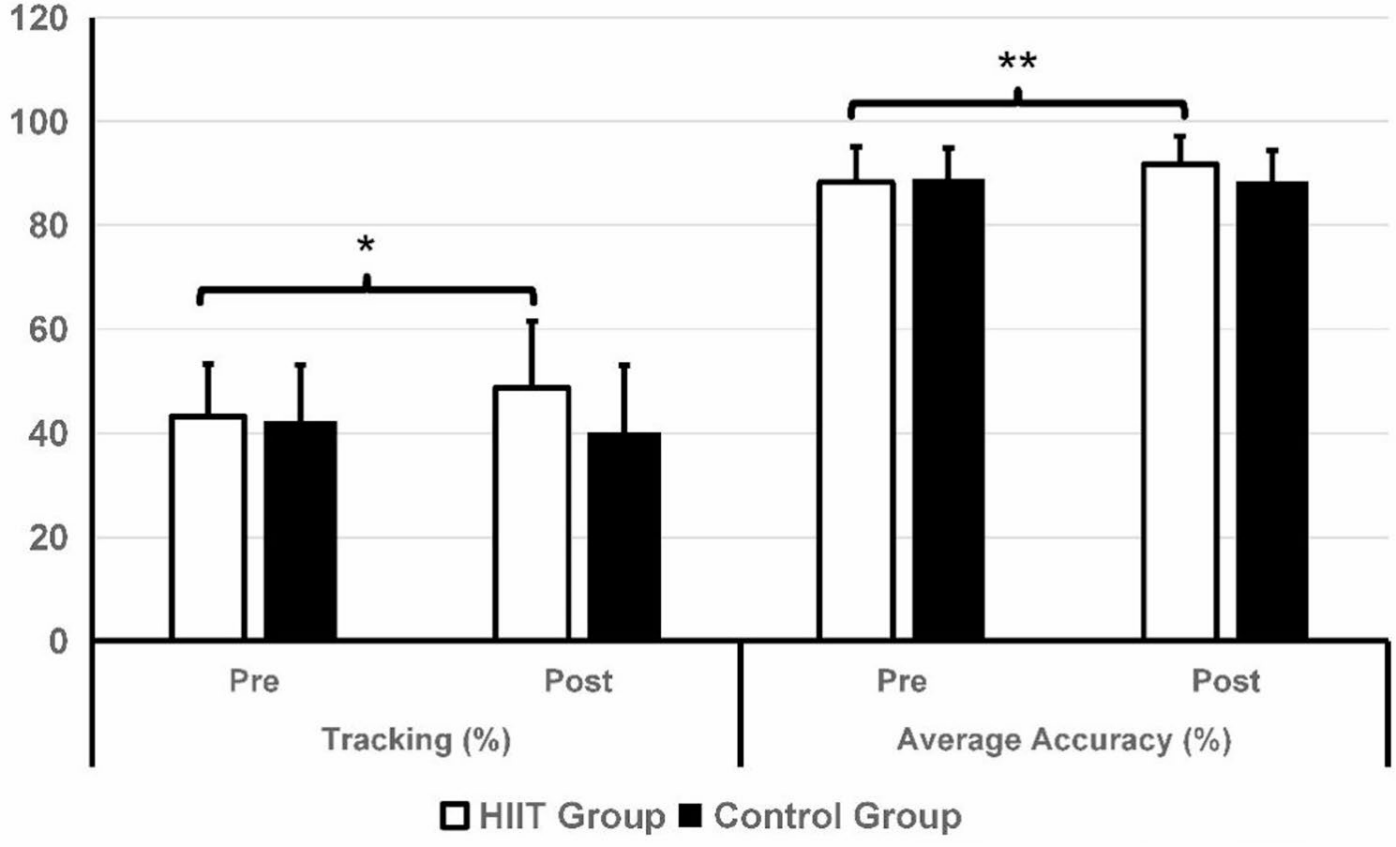
Comparison between the HIIT group and control group in the flicking and decision-making test. *p < 0.05, **p < 0.01

**Fig. 4:**
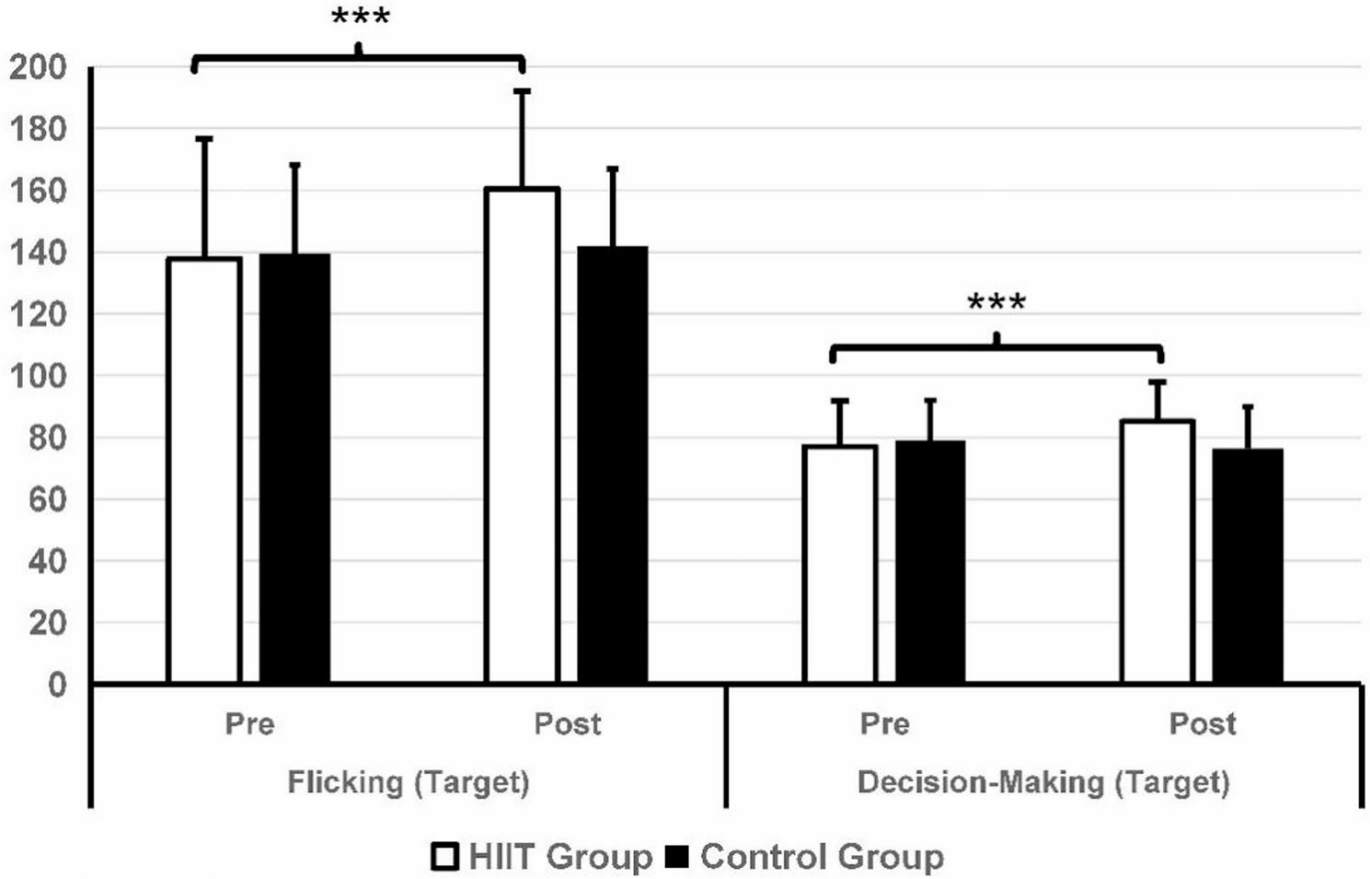
Comparison between the HIIT group and control group in the tracking test and shooting accuracy, ***p < 0.001

**Fig. 5:**
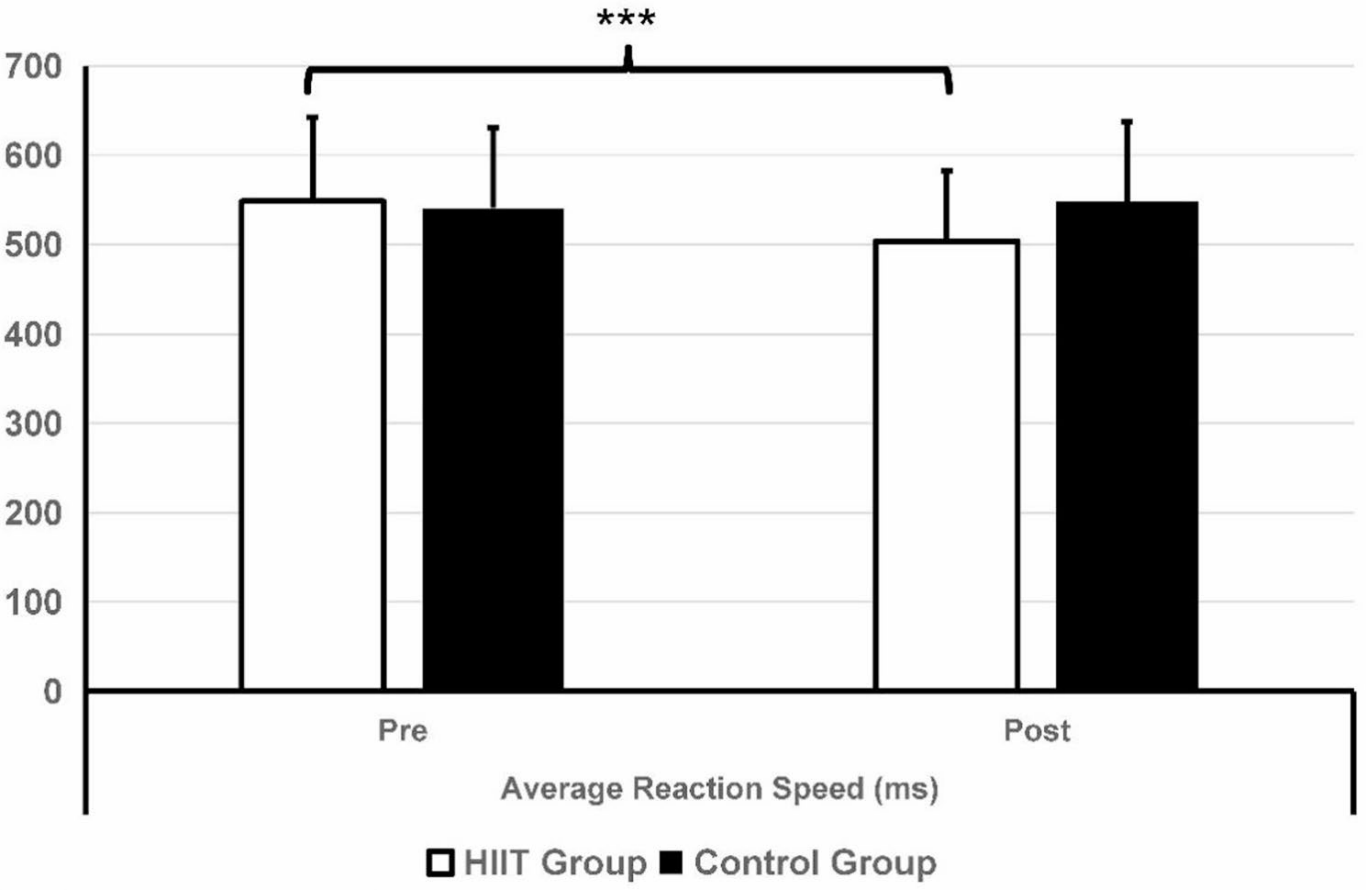
Comparison between the HIIT group and the control group in reaction time. ***p < 0.001

Table 3 lists the findings regarding esports performance. In the pre-test data, there were no significant differences between the two groups of players in terms of gameplay skills, working memory, mental toughness, and self-regulation (*p* > 0.05). Significant group-by-time interaction effects were found for flicking (*p* = 0.001), tracking (*p* = 0.005), decision-making (*p* < 0.001), accuracy (*p* = 0.01), reaction time (*p* = 0.001), and working memory (*p* = 0.01). After six weeks of HIIT training, as shown in Figs. 3 and 4, players in the HIIT group showed significant improvements in flicking (*p* < 0.001, t = 4.416, d = 0.69), tracking (*p* = 0.018, t = 2.468, d = 0.38), and decision-making (*p* < 0.001, t = 4.343, d = 0.68). In contrast, players in the control group did not exhibit any significant differences in their performance in the tests after six weeks. The intervention group demonstrated reduced reaction times (*p* < 0.001, Z = − 3.901) and higher shooting accuracy (*p =* 0.002, Z = − 3.085) compared to their performance six weeks prior (Figs. 4 and 5). No significant statistical differences in reaction time and accuracy were observed in the control group before and after the six-week period.

In the supplementary tests for working memory and mental toughness, a significant interaction effect of time and group was found in working memory, but no significant time-by-group interaction effects were observed for mental toughness and self-regulation. The intervention group showed a significant improvement in the Backward Corsi Task after six weeks (*p* = 0.002, Z = − 3.108). However, no significant improvements were observed in mental toughness and self-regulation. The control group did not show any significant differences in these three tests compared to their pre-test outcomes.

**Table 3:** Summary of the analysis of esports performance outcomes

Primary outcomes	HIIT group (*n* = 41)	Control group (*n* = 42)	*p*	Group-by-time interaction effect (*p*)
Flicking (Target)				
Pre	137.90 (38.76)	139.51 (28.77)	0.831 (t = − 0.214)	0.001
Post	160.48 (31.67)	141.90 (25.11)	0.004 (t = 2.965)	
*p*	< 0.001 (t = 4.416)	0.501 (t = 0.679)		
**Tracking (Rate**,** %)**				
Pre	43.20 (10.02)	42.36 (10.76)	0.713 (t = 0.369)	0.005
Post	48.72 (12.83)	40.17 (12.90)	0.003 (t = 3.028)	
*p*	0.018 (t = 2.468)	0.183 (t = − 1.358)		
**Decision-making (Target)**				
Pre	77.03 (14.78)	78.90 (13.11)	0.543 (t = − 0.610)	< 0.001
Post	85.14 (12.75)	76.43 (13.53)	0.003 (t = 3.017)	
*p*	< 0.001 (t = 4.343)	0.132 (t = − 1.536)		
**Average accuracy (Rate**,** %)**				
Pre	88.30 (6.78)	89.00 (5.96)	0.827 (Z = − 0.219)	0.01
Post	91.80 (5.38)	88.55 (5.94)	0.006 (Z = − 2.760)	
*p*	0.002 (Z = − 3.085)	0.255 (Z = − 1.138)		
**Average reaction speed (ms)**				
Pre	548.50 (93.98)	541.02 (90.18)	0.604 (Z = − 0.519)	0.001
Post	503.83 (78.99)	548.71 (88.98)	0.040 (Z = − 2.058)	
*p*	< 0.001 (Z = − 3.901)	0.071 (Z = − 1.807)		
**Working memory (Target)**				
Pre	5.82 (1.22)	6.05 (0.91)	0.427 (Z = − 0.795)	0.01
Post	6.55 (0.89)	6.02 (1.17)	0.019 (Z = − 2.346)	
*p*	0.002 (Z = − 3.108)	0.842 (Z = − 0.199)		
**Mental toughness (Score)**				
Pre	36.51 (4.24)	36.79 (4.29)	0.771 (t = 0.292)	0.531
Post	37.45 (3.88)	36.81 (3.88)	0.466 (t = − 0.732)	
*p*	0.353 (t = 0.939)	0.979 (t = 0.273)		
**Self-regulation (Score)**				
Pre	208.10 (18.69)	205.57 (22.58)	0.581 (t = − 0.554)	0.508
Post	204.86 (23.83)	198.03 (21.58)	0.175 (t = − 1.369)	
*p*	0.503 (t = − 0.676)	0.101 (t = − 1.675)		

###  Effect of the Exercise Intervention on the Secondary Outcomes

For measured secondary outcomes, we analyzed players’ weight, physical activity levels, sleep quality, VO_2max_, and affect. Table 4 presents the overall analysis results of these secondary outcomes. These metrics did not exhibit significant differences at baseline. No significant time-by-group interaction effects were found for weight and affect, and neither group exhibited significant changes over the six-week period. However, significant time-by-group interaction effects were observed in sleep quality, physical activity levels, and VO_2max_. After completing six weeks of HIIT, the exercise group showed significant improvements in sleep quality, physical activity levels, and VO_2max_, while no significant changes were observed in the control group. Furthermore, Spearman’s test indicated that changes in VO_2max_ were significantly correlated with changes in overall reaction time (*p* = 0.005, r_s_ = − 0.432).

**Table 4:** Summary of the analysis of secondary outcomes

Secondary outcome	HIIT group (*n* = 41)	Control group (*n* = 42)	*p*	Group-by-time interaction effect (*p*)
Weight (kg)				
Pre	66.77 (10.62)	68.37 (14.33)	0.863 (Z = − 0.137)	0.381
Post	67.42 (9.67)	67.68 (12.94)	0.792 (Z = − 0.792)	
*p*	0.216 (Z = − 1.237)	0.118 (Z = − 1.557)		
**Physical activity (MET-min/w)**				
Pre	2077.65 (1567.54)	2385.59 (1861.84)	0.569 (Z = − 0.569)	0.014
Post	2647.59 (2005.58)	2141.12 (1601.74)	0.238 (Z = − 1.180)	
*p*	0.01 (Z = − 2.559)	0.425 (Z = − 0.797)		
**Sleep quality (Score)**				
Pre	5.01 (2.63)	5.45 (1.90)	0.216 (Z = − 1.236)	0.040
Post	4.11 (1.98)	5.53 (1.60)	< 0.001 (Z = − 3.540)	
*p*	0.044 (Z = − 2.014)	0.908 (Z = − 0.116)		
**VO**_**2max**_ **(mL/kg/min)**				
Pre	28.44 (6.97)	28.66 (6.20)	0.881 (t = − 0.150)	< 0.001
Post	33.81 (6.99)	28.42 (5.66)	< 0.001 (t = 3.868)	
*p*	< 0.001 (t = 7.292)	0.665 (t = − 0.117)		
**Positive affect (Score)**				
Pre	26.63 (9.049)	24.17 (7.22)	0.173 (t = 1.375)	0.464
Post	28.02 (7.273)	24.51 (6.96)	0.028 (t = 2.243)	
*p*	0.209 (t = 1.227)	0.714 (t = − 0.370)		
**Negative affect (Score)**				
Pre	14.68 (4.447)	15.17 (3.93)	0.392 (Z = − 0.857)	0.936
Post	14.45 (3.758)	15.01 (5.38)	0.978 (Z = − 0.027)	
*p*	0.877 (Z = − 0.154)	0.406 (Z = − 0.832)		

### Perceptions of Players in the HIIT Group

Table 5 provides a categorized summary of the thematic analysis of player interviews, highlighting the key themes related to game performance and non-game-related improvements following a six-week HIIT program. The themes are further divided into sub-themes to provide a comprehensive understanding of the players’ subjective experiences. The majority of participants reported perceived enhancements in their gaming performance metrics following sustained physical exercise engagement, specifically noting improved concentration and reduced gaming-related fatigue. Beyond performance metrics, our findings indicate that most participants perceived positive shifts in their gaming mentality and demonstrated increased optimism. As one participant noted, “*I find myself less prone to anger when losing games*,* and I remain more composed during unfavorable game situations. I’m also less likely to let toxic behavior from teammates in the game affect my real life*”.

**Table 5:** Summary of themes in players’ perceptions of the six-week HIIT program

Category	Main theme	Sub-themes
Game-related	In-game performance	-Enhanced game statistics (e.g. more kills, improved game rank, higher win rate) -Faster reaction time -Higher hit rate
	Game-related abilities	-Better concentration -Increased emotional stability and calmness -Reduced game stress -Enhanced game mindset -Decreased gaming fatigue -Less affected by negative emotions in games
Non-game related	Better physical health	-Improved overall fitness and stamina -Increased vitality and energy level -Greater motivation to exercise
	Improved mental well-being	-More relaxed and stable emotions -Improved mood -Better regulation of daily routines
	Enhanced sleep quality	-Improved sleep quality -Easier sleep onset

## Discussion

This study investigated the effects of a six-week HIIT intervention on the esports performance of young adult FPS players. The results demonstrate that HIIT training (three times per week) led to significant improvements in multiple aspects of gaming performance: flicking (16.4%), tracking (12.8%), decision-making (10.5%), shooting accuracy (4.0%), reaction time (− 8.1%, − 44.7ms), and working memory (12.5%). However, no significant improvements were observed in players’ mental toughness and self-regulation measures. Furthermore, the HIIT intervention positively impacted players’ daily physical activity levels, VO_2max_, and sleep quality. The changes in VO_2max_ over the six-week intervention period were moderately correlated with changes in game reaction times.

Although many previous studies have found that a single session of HIIT significantly improves players’ game performance [14, 41], further empirical evidence is still needed to confirm the sustained effects of multiple exercise interventions. Our findings present some contrasts with a recently published experiment on exercise intervention’s effects on short-term esports performance [61]. Although both studies utilized some similar tests in Aim Labs, they differed in chosen performance metrics, sample sizes, and participant characteristics. Notably, the participants in that study reported longer weekly gaming hours and gaming history, suggesting that relatively novice players might be more responsive to exercise intervention. Furthermore, the mean age of our participants was approximately 5–6 years younger than that of their study population, indicating that gaming performance adaptations and enhancements may be more evident in younger cohorts. Additionally, the varying responses to exercise intervention might be attributed to differences in baseline physical activity levels among players, as individuals with different exercise backgrounds may exhibit different sensitivities to exercise-induced adaptations [88, 89]. Another significant distinction lies in the different exercise protocols adopted by the two studies, particularly in terms of the stringency of monitoring measures, which might have affected the quality of HIIT completion and adherence. These factors may collectively account for the inconsistent results in long-term intervention studies. Given the current scarcity of research in this field, these findings still require validation through more long-term studies in the future. These methodological differences and participant characteristics highlight the complexity of exercise intervention effects on esports performance and emphasize the need for more standardized research protocols in future studies.

Based on the existing research on HIIT, game performance, and cognitive function, several potential mechanisms can be proposed. First, HIIT has been shown to improve cardiovascular health and VO_2max_, which may enhance cognitive functions by increasing cerebral oxygenation [90]. A recent pre-post experimental study on elite esports players also observed significant alterations in cerebral hemodynamics, executive function, and heart rate variability following a 10-week exercise intervention [17]. Second, HIIT may improve game performance by reducing stress and enhancing mood, which aligns with the experience and perception of players participating in HIIT in this study [91, 92]. Previous research has indicated that physical training can improve mood and cognitive function by modulating neurotransmitter levels and reducing inflammation [93]. Furthermore, the positive effects of HIIT on game performance may also be related to its neurophysiological impacts. Studies have shown that HIIT can promote brain health through various mechanisms, including increasing levels of brain-derived neurotrophic factor (BDNF), promoting neurogenesis, and enhancing synaptic plasticity [94, 95]. These changes may improve cognitive processing speed and efficiency, thus enhancing reaction times and decision-making abilities in games. Future research could employ neuroimaging techniques such as functional magnetic resonance imaging (fMRI) and electrophysiological recordings to explore how HIIT affects brain function and game performance [31, 96]. Additionally, future esports performance research could also incorporate objective physiological monitoring indicators like stress response, such as salivary cortisol and heart rate variability, to further explore these physiological mechanisms.

While HIIT has shown significant benefits in enhancing game performance, other types of physical training, such as strength training and flexibility training, may differentially influence psychological and cognitive abilities. Strength training can enhance muscle strength and endurance, improving physical control and stability, which are crucial in games requiring fine motor control [10, 19]. Similarly, flexibility training may contribute to overall body coordination and reduce gameplay errors [11, 97]. The differential effects of these training modalities on gaming performance warrant further investigation through comparative studies.

A notable finding from our study was the significant improvement in participants’ sleep quality following the six-week intervention. Given the well-established correlation between sleep quality and cognitive function [98, 99], this improvement may have indirectly enhanced players’ esports performance. The sleep-performance relationship in esports contexts merits further investigation, particularly considering the irregular sleep patterns often observed in competitive gamers. The results of the analysis on VO_2max_ are consistent with previous research demonstrating the benefits of HIIT on improving VO_2max_ and the correlation between VO_2max_ and cognitive function. However, the results differ concerning mental toughness and self-regulation [100, 101]. This discrepancy might be due to differences in measurement tools and methods used in various studies, which might also indicate that these psychological traits require longer or more diverse interventions for improvement. However, the intensity and duration of the HIIT protocol implemented in this study were specifically tailored for participants without prior regular exercise habits, resulting in relatively lower intensity and shorter duration. Consequently, this may have limited our capacity to detect potential enhancements in mental toughness and self-regulation attributable to HIIT in the present investigation [102]. Future research should explore varying exercise protocols and potentially incorporate specific psychological training techniques to better understand and enhance these aspects in esports players.

The qualitative component of our study, involving exploratory interviews with participants, revealed several consistent themes: improved focus during gameplay, extended concentration capacity, enhanced emotional stability, reduced gaming-related fatigue and stress, and a more positive mindset. While these subjective experiences require validation through empirical studies, they provide valuable insights for future research directions and practical applications in esports training programs.

The implications of our findings extend beyond immediate performance benefits, suggesting that incorporating HIIT into esports training routines could create a paradigm shift in how we approach esports player development. Several health-related concerns have been traditionally associated with excessive screen time and prolonged sedentary behavior in video gamers [103, 104]. Studies have linked prolonged screen time with lower levels of physical activity and poor cardiovascular health [105, 106]. Except for active video games or virtual sports, the majority of video games are typically played in a sedentary position [107]. Therefore, excessive sitting time and screen exposure pose potential risks to the health of esports athletes. In this context, integrating HIIT into esports training routines addresses both performance enhancement and health risk mitigation. Firstly, HIIT can help mitigate the cardiovascular health risks associated with prolonged sedentary time in professional esports players [108]. This finding supports the argument for encouraging sedentary gamers to change their physical activity habits. Additionally, previous studies have found that participation in esports may be associated with increased motivation for physical exercise [109, 110], and high-level esports athletes often exhibit higher levels of physical activity [111]. This suggests a positive feedback loop where physical exercise and esports performance mutually reinforce each other, creating opportunities for holistic athlete development. This also challenges the conventional impression that physical activity and video gaming are typically regarded as non-complementary and time-competing activities [112].

By promoting a balanced lifestyle that includes regular physical activity, esports organizations could enhance the overall well-being and performance of their players. This holistic approach not only benefits the physical health of players but also contributes to their mental and emotional stability, which are critical components of sustained high-level performance in esports. Additionally, exploring the potential synergistic effects of combining physical exercise with other performance-enhancing strategies, such as nutritional interventions and cognitive training, could yield comprehensive insights into optimizing esports performance. The use of advanced biometric monitoring and neuroimaging techniques could further elucidate the underlying mechanisms through which physical exercise influences cognitive and psychological outcomes in esports players.

### Limitations

The sample size was relatively small, lacking representation from top-tier professional players and female players. Furthermore, due to the regulatory constraints in China, this study did not include minors. Additionally, this study only included participants lacking regular exercise habits, and whether esports players who already maintain regular physical activity would show similar responsiveness to and derive significant benefits from HIIT interventions of this intensity and frequency remains to be explored. The significant sex imbalance in our sample reflects both the current male-dominated nature of competitive gaming and the broader challenges and barriers that female players might face in esports [113]. This lack of sex diversity, to some extent, also limits the generalizability of our findings. Future research with larger sample sizes could further analyze the differential effects of HIIT interventions across various esports and physical activity levels, sexes, and ages among esports players.

In terms of measurement, this study used proxy tests for VO_2max_ assessment and relied on self-reported measures for some indicators, which may introduce certain errors. Future research could employ more objective testing methods to obtain more accurate measurements. Additionally, although Aim Labs simulated most scenarios and assessed core abilities required in FPS games, it still cannot fully represent the strategic decision-making that players face in actual games. Future research needs to develop more scientific and comprehensive methods for measuring gaming performance.

Given that many game players in this age group are students whose lifestyles and gaming habits can change significantly over time, it is challenging to conduct longer-term intervention studies that meet experimental settings. Consequently, the duration of this study was relatively short. Further exploration of integrating long-term physical training into esports training necessitates extended research periods and continuous monitoring of subsequent effects.

## Conclusion

This randomized controlled trial provides the first evidence that sustained and regular physical exercise interventions can significantly enhance esports players’ game performance, sleep quality, and VO_2max_. Notably, a moderate correlation was identified between changes in VO_2max_ and reaction times over the six weeks. However, no significant improvements were observed in mental toughness and self-regulation. These findings suggest that integrating physical exercise, particularly HIIT, into long-term esports training regimens has the potential to optimize performance and well-being. This research represents a pioneering step towards a comprehensive approach to esports training by incorporating physical exercise to enhance performance and health, especially as the field continues to professionalize rapidly. The implications of these findings extend beyond the development of individual players and have the potential to influence the standard practices of esports organizations worldwide.

## Data Availability

The complete datasets generated and/or analyzed during this study are not publicly available due to privacy protection regulations concerning participants’ personal information, but are available from the corresponding author upon reasonable request with appropriate data protection agreements in place.

## Abbreviations

BDNF: Brain-derived Neurotrophic Factor
DPI: Dots Per Inch
FPS: First-Person Shooter
GEE: Generalized Estimating Equations
HIIT: High-Intensity Interval Training
HR_max_: Maximum Heart Rate
IPAQ: International Physical Activity Questionnaire
PACER: Progressive Aerobic Cardiovascular Endurance Run
PANAS: Positive and Negative Affect Schedule
PAR-Q: Physical Activity Readiness Questionnaire
PSQI: Pittsburgh Sleep Quality Index
RPE: Rating of Perceived Exertion
SMTQ: The Sports Mental Toughness Questionnaire
SRQ: The Self-Regulation Questionnaire
VO_2max_: Maximum Oxygen Consumption
